# A comparative evaluation of loading times and exposures for permanent prostate brachytherapy

**DOI:** 10.1120/jacmp.v3i4.2550

**Published:** 2002-09-01

**Authors:** W. S. Bice, E. S. Walker, S. Gearty, A. V. Walker, J. R. Marbach, B. R. Prestidge

**Affiliations:** ^1^ University of Texas Health Science Center San Antonio Texas 78248; ^2^ International Medical Physics Services San Antonio Texas 78259; ^3^ Cancer Therapy Research Center San Antonio Texas 78248

**Keywords:** prostate, brachytherapy, iodine, radiation safety, needles

## Abstract

The loading of needles for loose seed implantation of the prostate gland results requires a significant amount of effort and some radiation exposure to members of the medical staff. This study was performed to quantify the time spent and exposure levels associated with implant preparation, as well as to investigate any improvement in the time or exposure burden due to the introduction of a new loading device. The movements and radiation exposures for two single, highly experienced dosimetrists were monitored for ten conventionally loaded iodine implant cases. These same cases were reloaded with dummy sources using the sleeved system to determine time savings, if any. Two of these ten cases were then loaded with live sources using the sleeved system to determine relative exposure to the loading staff between the two methods. The results were then analyzed to generate per‐seed and per‐needle loading time and exposure burdens. Formulas are presented that may be used to determine the average time to load implants and the resultant staff exposure, both with the conventional technique and with the sleeved method. On the average, it takes an experienced loader 48 min to prepare an implant for the operating room, receiving a hand dose of about 10 mrem and a whole body dose of about 1 mrem. The sleeved system reduced these values by at least half. The time and exposure burden associated with the preparation of iodine loose seed implants has been characterized. The use of the sleeved needles resulted in significant time and exposure reductions for the medical staff.

PACS number(s): 87.53Jw, 87.53.Xd

## INTRODUCTION

This year, in the United States alone, practitioners will perform more than 40 000 brachytherapy implants for the treatment of early stage prostate cancer.^1^ The popularity of this procedure stems not only from its efficacy and the low complication rate, but also because permanent prostate brachytherapy (PPB) corresponds to a savings in time and money for the patient and the medical community when compared to competing treatment modalities. PPB has, however, resulted in added burden to the medical staff, certainly in terms of additional radiation exposure, but also in the time required to prepare for the procedure.

Prostate implants are performed with either afterloading applicator devices or with preloaded needles and loose seeds. While superiority disputes abound between practitioners of these two techniques, one valid argument for the preloaded needle technique is the shortened operating room time. A portion of the time and exposure burden has been shifted out of the operating room by preloading each needle prior to the implant. Depositing the seeds within each needle is performed with a single, swift motion, shortening the overall implant time and reducing radiation exposures to the operating room staff. Unfortunately, preloading the needles results in an increased exposure and an added time commitment to the support staff that prepares the implant. This time commitment and radiation exposure from permanent prostate brachytherapy has never been the subject of published study. Previous radiation protection studies have concentrated on doses to members of the general public after the implant has been performed.^2^ ^–^ ^4^

**Figure 1:**
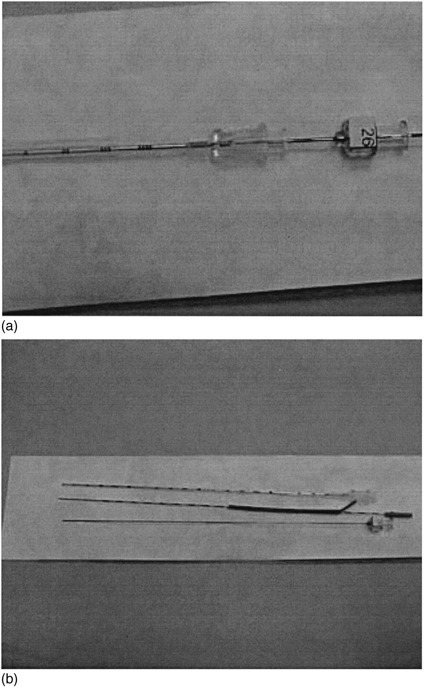
(a) The Imagyn isosleeve™ needle shown with, from left to right, the needle, sleeve and stylette sheathed. (b) The isosleeve™ system with each component above displayed separately.

Various seed manufacturers have addressed the problems presented by preloading needles. Time and exposure reduction are worthy goals, certainly form the perspective of the implant loader. Solutions have ranged from cartridge systems to automated loading devices. Recently Imagyn Medical Technologies, Inc., (Irvine, California) has developed a preloaded needle packaging and delivery system (isosleeve™) that promises to offer several advantages over conventional loose seed loading. In this system, the user orders the needles in a configuration designed for the implant. These needles arrive sterile and preloaded, with a record depicting the needle‐loading pattern. The loading can be visually checked because a transparent sleeve containing the sources and spacers can be pulled from the needle, inspected and reinserted. We obtained loose seeds with each implant to be able to perform an independent assay. After assay these loose seeds are sterilized and then hand loaded into additional needle assemblies provided with the order. Pictures of this source delivery system is shown in (Figs. 1a) and (1b).

The purpose of this study was twofold: first, to quantify the time investment and radiation exposure of the loading staff in conventionally loaded loose seed procedures; second, to determine any time or dose equivalent savings that might be realized in using the sleeved system.

## MATERIALS AND METHODS

Over a three‐week period, we chose to study ten consecutive I125 implant cases. These ten cases were loaded conventionally using the preloaded needle technique. The same ten cases were reloaded and studied using the sleeved system and demonstration, or dummy, seeds. Two of the cases were then reloaded using live seeds and the sleeved system. The source strength for each implant was chosen to match the anisotropically corrected dose rate of 0.4092 cGy/hr at 1 cm from the source using the point source approximation formalism.^5^ The average number of sources and needles for these cases, by type, is given in Table I.

**Table I:** Averages over all cases of each type. The numbers in parentheses are the standard deviations. P values (two‐tailed T‐test, paired data) ≤0.05 are listed to show statistically significant differences. All times (values below the double line) are in s (except the last row).

Implant characteristics and loading times	Average (conventional loading, including survey)	Average (sleeved loading, including survey)	*P* value
Seeds/implant	95.6 (14.4)	86.0 (12.8)
Needles/implant	26.40 (2.7)	24.0 (2.7)
Live seeds (10 and 2 cases)	95.6 (14.4)	86.5 (16.3)
Dummy seeds (10 cases)		85.9 (13.1)
Open box (s)	42.1 (7.8)	57.4 (18.2)	0.044
Verify calibration certificate (s)	141.6 (63.9)	76.6 (24.7)	0.007
Log in seeds (s)	63.4 (23.2)	45.0 (7.6)
Count seeds (s)	247.7 (110.8)	39.7 (13.6)	<0.001
Prepare for assay (s)	70.5 (48.6)	23.0 (7.6)	<0.015
Source assay averages (s)	^a)^	^a)^
Wrap for sterilization/survey (s)	54.2 (12.3)	47.7 (15.4)
Set up sterile field (s)	30.9 (6.8)	31.9 (19.7)
Open packages (s)	126.0 (25.7)	78.6 (26.2)	<0.001
Open sterile packages (s)	176.26 (28.4)	82.4 (14.7)	<0.001
Plug needles (s)
Layout seeds (s)	277.2 (55.4)	34.2 (11.3)	<0.001
Needle loading averages (s)	^b)^	^b)^
Wrap up case and survey (s)	123.2 (48.0)	57.8 (26.3)	0.006
Total time (seconds)	2880.6 (609.0)	1223.0 (331.4)	<0.001
Total time (minutes)	48.0 (10.2)	20.4 (5.5)	<0.001

a) Source assay times. The average total source assay time was given by (44.2)(S), where S is the number of seeds and the result is in s.

b) Needle loading times are shown in Figs. 5 and 6.

The tasks associated with the preparation of each case for implantation are also listed in Table I. Software was developed to track and time the performance of these tasks and to record notes for each case. Exposure rate readings (Ludlum Model 3 detector with model 44 pancake probe) were recorded for counting seeds and for organizing the seeds for needle loading. The total exposure was calculated by obtaining the product of the exposure rate and the time for the task. For tasks that included only a portion of the seeds, the exposure rate was adjusted in a linear fashion for the number of sources contributing to the dose at the time of exposure. For instance, if the exposure rate was measured when there were 100 seeds present and the task was timed when there were only 30 seeds present, the exposure rate was adjusted by multiplying by (30/100) or 0.30. An energy correction factor of 1.8 (from the energy response curve of the detector) was applied to the readings to correct for the low energy iodine spectrum. Thermoluminescent dosimeters (TLD), a ring badge (Landauer, minimum detectable reading 30 mrem), and a whole body badge (Landauer, minimum detectable reading 1 mrem), were also worn for each case in which live seeds were used. The integrated dose that was calculated from the exposure rate measurements and the time study was compared to the integrated dose from the TLD.

A Standard Imaging Model IVB 1000 Reentrant Well Chamber was used to assay both the conventional loading and the sleeved cases. Individual seeds were assayed using the single seed insert. NIST‐traceable calibration factors were provided by an accredited dosimetry calibration laboratory (ADCL) and were verified using a single, calibrated source procured from the manufacturer.

The task list that was used is shown in Table I. With the exception of survey and swipe testing the shipment upon receipt and the actual sterilization time, these are all of tasks that must be performed in order to prepare a case for the operating room. The survey and swipe tests at our institution are performed prior to our receipt of the shipment. This was measured one time, performing these tasks with the associated record keeping took 86 s, excluding the swipe counting time.

The actual sterilization time varies widely between institutions, dependent upon local sterilization protocols. The staff that performs this task also varies. At our institution the dosimetrist uses a steam autoclave to heat the sources to 270° for 3 min to perform the sterilization. The data presented in this study does not include survey and swipe testing or sterilization time.

Note that plugging of needles with bone wax is not performed with either technique. We use preplugged needles for conventional loading and the sleeved system requires no plugging. Earlier, unpublished, work indicates that the time required to perform this task averages about 128 s.

We chose to visually verify the loading of each sleeved needle. With the presence of the record, some may consider this act superfluous, but the time and exposure burden is included in this study for those who would find it prudent. The overall time and exposure comparisons between the two methods includes this inspection step.

**Figure 2:**
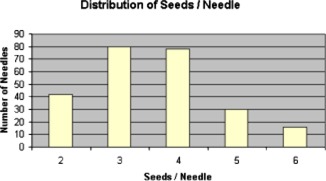
(Color) The distribution for the needles used for all of the cases in this study classified by the number of sources loaded into each needle.

Our average implant for this series consisted of 96 seeds in 26 needles. The distribution of needles with different numbers of seeds for all implants is shown in Fig. 2. The average number of seeds per needle was 3.59.

## RESULTS

The average times for each of the tasks listed in the task list are also shown in Table I. The first column is for conventionally loaded implants; the second shows the average for the sleeved cases. Standard deviations are listed in parentheses behind each value. Because the data was paired, we were able to use a Student's *t*‐test to derive a statistical comparison between the conventional and sleeved timings. This value is indicated in the table only for task‐timing differences that were significant (p<0.05).

**Figure 3:**
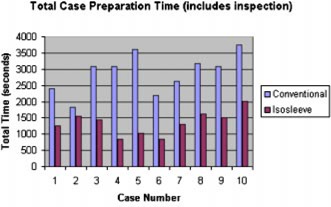
(Color) Total time for the ten cases studied. Conventional and sleeved cases for the same patient are shown side by side.

Figure 3 shows a side‐by‐side comparison of the total time required to prepare each case, conventionally and with the sleeved system. In every case there was a time saving realized in the sleeved cases. Table I shows that the average time dropped from 48 to 20 min.

Assay preparation times were different between the two systems largely because of the number of seeds available for assay. With the sleeved system we asked for 10% of the seeds to be sent loose, available for assay and hand loading. The overall assay time was dependent upon the number of sources to be assayed. Although there was a slight decrease in the assay time per source as the assay progressed, the difference was small enough, 7–8 s on the average, that we felt that a simple model of source assay was justified. The assay time for the sources was then shown to be

(1) $$T_{assay}=(44.2)(S_{A}),$$

where $S_{A}$ is the number of sources assayed and the result is in seconds.

**Figure 4:**
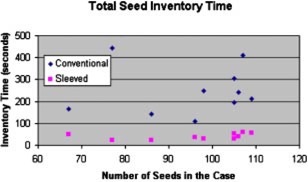
(Color) Inventory (counting) time for both conventional and sleeved cases plotted against the number of sources in each case. Average total values for each were used in the model due to the lack of an apparent relationship.

The seed inventory, listed as “Count Seeds” in the task list, would also be expected to be dependent upon the number of sources. As shown in Fig. 4, this is not the case. No trend was noted for either the conventional or the sleeved cases. The inventory time was substantially reduced (p<0.001) for the sleeved cases however, due to the organization inherent in the packing.

The same relationship was noted for organizing or laying out the seeds in preparation for loading. There was almost no dependency upon the number of sources and the sleeved case times were substantially lower. This was due of course to the reduced number of seeds to be hand loaded in the sleeved cases.

**Figure 5:**
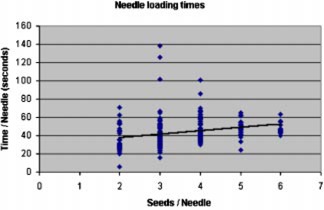
(Color) The relationship between the number of sources in the needle and the loading time for conventional loading of needles. The equation for the fitted line is used in the conventional model and in the sleeved model for the hand loaded needles.

Figure 5 shows the relationship that exists between the number of sources per needle and the time it takes to load a needle by hand. The standard deviation for each of the data points (two seeds, three seeds, etc.) is quite large. Upon review of the data, it became apparent that the instances during the loading of a case that a seed or spacer jammed in the needle hub during conventional loading contributed significantly to the variance in loading times.

Needle loading can be modeled with an overhead term and a cost term dependent upon the number of seeds per needle,

(1) $$T_{needle\;loading}=\Sigma_{N}[30.2+3.72(S_{N})]=30.2(N)+3.72(S_{T}),$$

where

*T* is the total needle loading time (s),

*N* is the number of needles,

$S_{N}$ is the number of seeds in needle,

$S_{T}$ is the total number of sources in the implant. The summation is made over all of the needles, *N*.

The time associated with the preparation of a loose seed implant can then be characterized by an equation dependent upon the number of seeds and needles. The equation consists of an “overhead” term to account for tasks like receipt of the shipment, packaging the implant for sterilization, and other tasks independent of the size of the implant. In reality there is still some dependence of the overhead terms on the total number of sources in the implant. A linear least square error fit of the overhead timings can be modeled as

(3) $$T_{overhead}=1127.7+(2.17)(S_{T}).$$

Combining the three terms, overhead, assay and needle loading gives the following:

(4) $$T_{conv}=1127.7+(2.17)(S_{T})+(44.2)(S_{A})+\Sigma_{N}[30.2+3.72(S_{N})],$$

where $T_{\text{conv}}$ is the total time for conventional loading (s).

If we assume that the number of seeds assayed is the total number of seeds divided by 10 and, from Fig. 2, the distribution of seeds per needle is the average seeds per needle, 3.59, Eq. (4) reduces to

(5) $$T_{conv}\approx 1127.7+(18.72)(S_{T}).$$

An analogous equation used to model the sleeved cases included a needle inspection time, (17.86)$\left(N_{\text{sleeve}}\right)$, where $N_{\text{sleeve}}$ is the number of sleeved needles in the case.

If the same assumptions with regard to the number of assay sources and average seeds per needle are made, the approximate time for preparation of the sleeved cases becomes

(6) $$T_{sleeve}\approx 265.2+(11.34)(S_{T}).$$

**Figure 6:**
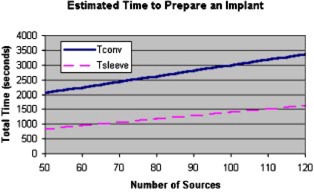
(Color) Total loading times determined by the models for conventional and for sleeved loading.

Figure 6 shows the relationship of the total loading times for the estimates presented by the equations above. Both the overhead term and the time invested per seed are lower in the sleeved case, confirming the result shown in the raw data—the time was shortened in the sleeved loadings for every implant.

We used a very simple model to determine the radiation exposures to the staff. We use a leaded acrylic sheet to shield the loader from the sources and reverse action tweezers to handle the seeds. By direct measurement of the exposure rate of the radiation field at the level of the hands we found that the average dose equivalent to the hands when the seeds were laid out was about $28\;\text{mrem}\;{\text{hr}}^{-1}$ and $0.1\;\text{mrem}\;{\text{hr}}^{-1}$ to the collar badge for the 109 seed case. We assumed in this model then that the dose equivalent to the hands could be expressed as $28/109=0.257\;\text{mrem}{\text{hr}}^{-1}=7.14\times 10^{-5}\;\text{mrem}\;s^{-1}$ for each seed near the hands during the loading process. Similarly, the collar badge would see a dose equivalent rate of about $0.001\;\text{mrem}\;{\text{hr}}^{-1}{\text{seed}}^{‒1}=2.78\times 10^{-7}\;\text{mrem}\;s^{-1}$. The staff exposure was estimated from the product of the exposure rate measurements and the time‐study results and then compared to the TLD measurements.

Most of the dose to the hands in conventional loading comes from counting the seeds, laying out the seeds, and loading the needles. Our model for the dose then becomes

(7) $$D_{hands,\;conv},=7.14\times 10^{-5}(S_{T})(T_{count}+T_{layout})+\Sigma_{N}(D_{0})(S_{out}/S_{T})(t_{N}),$$

where

$D_{\text{hands,conv}}$ is the dose equivalent to the hands (mrem),

$T_{\text{count}}$ is the time taken to count the seeds,

$T_{\text{layout}}$ is the time taken to organize or layout the seeds,

$D_{0}$ is the exposure reading at the beginning of the needle loading $\left(\text{mrem}\;s^{-1}\right)$,

$S_{\text{out}}/S_{T}$ is the fraction of sources still laid out when loading needle, *n*,

$t_{N}$ is the time taken to load needle, *N*.

For our study, where the count and layout times were relatively insensitive to the number of seeds, this expression reduces to

(8) $$D_{hands,\;conv},=0.0375(S_{T})+\Sigma_{N}(D_{0})(S_{out}/S_{T})(t_{N}).$$

This may be further simplified if the assumption is made that the needle loading occurs so that the number of seeds laid out at any time during the loading decreases linearly with time, i.e.,

(9) $$N_{t}=S_{T}-(S_{T})(t/T_{load}),$$

where

$N_{t}$ is the number of seeds laid out at any time, *t*,

$T_{\text{load}}$ is the total time it takes to load the seeds into the needles (*s*).

The summation in the second term of Eq. (8) can then be evaluated as an integral over the needle loading time, which then reduces to $D_{0}T_{\text{load}}/2$. The time to load a needle has already been shown to be a function of the number of seeds per needle in Fig. 5. If we further assume that the distribution of sources per needle in each case remains that shown in Fig. 2, the average seeds per needle is 3.59. From Eq. (2), this average can then be used to determine the total needle loading time as (43.6)(*N*), where *N* is the number of needles, or (12.1)$\left(S_{T}\right)$, where $S_{T}$ is the number of seeds. Under these assumptions, Eq. (8) may be further reduced to

(10) $$D_{hands,\;conv}=(0.0375)(S_{T})+(12.1)(D_{0})(S_{T}).$$

The geometry that we used to load needles resulted in an average $D_{0}$ to the hands was about $7.14\times 10^{-5}\;\text{mrem}\;s^{-1}\;{\text{seed}}^{-1}$. This gives an estimate of

(11) $$D_{\text{hands},\;\;\text{conv}}=0.0375(S_{T})+8.64\times 10^{-4}{\left(S_{T}\right)}^{2}$$

Based upon measured exposure rates, the whole body dose was assumed to be 0.004 times the dose to the hands when the operator was working behind the acrylic *L*‐block shield. This was obviously too conservative as the loader cannot realistically keep the *L*‐block between himself and the implant the entire time. From the average TLD data we adjusted this value to 0.10 of the dose to the hands.

Two exposure differences exist for the sleeved cases as opposed to the conventional cases. For the sleeved cases, the hand loading procedure is reduced to the 10% of the seeds that were held out for assay, reducing the number of conventionally loaded needles by a factor of about 10. However, the inspection of each needle to verify the loading increases with the exposure because of the inspection time. We have assumed that the dose to the hands during needle inspection consists of the dose equivalent rate for each seed, $7.14\times 10^{-5}\;\text{mrem}\;s^{-1}$; times the number of seeds inspected in this fashion, (0.9)($S_{T}$); times the time required to inspect each needle, 17.86 s. This results in the equation for sleeve‐loaded case preparation doses,

(12) $$\begin{array}{ll} D_{\text{hands},\;\text{sleeve}} & =(0.0375)(S_{T})+(0.1)(0.1)(8.64\times 10^{-4}){\left(S_{T}\right)}^{2}+(7.14\times 10^{-5})(17.86)(0.9)\\ & =(0.0386)(S_{T})+(8.64\times 10^{-5}){\left(S_{T}\right)}^{2} \end{array}$$

**Figure 7:**
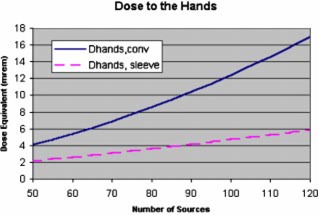
(Color) Dose to the hands as calculated from the conventional and sleeved models.

The dose to the hands is plotted for comparison between conventional and sleeved loading in Fig. 7. Using the same arguments as for conventional loading, we have adopted the same factor‐of‐ten rule for whole body exposure from the sleeved cases: the whole body dose is a factor of 10 less than the dose to the hands.

In compiling the TLD results we only used the readings that showed above the minimum detectable level. This occurred for four whole body badges and for seven ring badges. Excluding the badges below the minimum detectable level artificially raises the exposure estimates; including these readings would have artificially lowered them. Therefore, the TLD data, Table II, should be considered as worst‐case estimates. Except for one reading, 7 mrem versus 9 mrem, the deep and shallow doses were the same for all of the whole body badges.

**Table II:** Average exposures for the monitored cases. Only cases where the dose equivalent was greater than the minimum detectable by the TLD were included in the results.

Type	Extremity exposure (mrem)		Whole body exposure (mrem)	
	Dose rate‐time (10 cases)	TLD (7 cases/2 cases)	Dose rate‐time (10 cases)	TLD (4 cases/2 cases)
Conventional	11.6	41.4	1.2	4.5
Sleeved	4.5		0.5	

The TLD showed no correlation between dose equivalent, either to the hands or to the whole body, with either the number of seeds or the product of seeds and case preparation time, as suggested by Eq. (7).

## DISCUSSION

Each institution performs implants differently. We present the results of this study, characterizing the time and dose burden from loose seed implants, as an example of that expected for an active, medium‐sized program. Both of our loaders were highly experienced; we would expect the results to change for less experienced medical staff or for programs that prepare their implants differently or with different equipment.

While it was obvious that use of the sleeved system saved a considerable amount of time and reduced the radiation exposure, there are areas of possible improvement. A shield system provided with the drape containing the needles would lower the dose. An assay insert that could be sterilized would save a considerable amount of time, not only during the assay procedure, but also eliminating the need to hand load 10% of the seeds into needles. Needles labeled according to the template location, as opposed to sequential numbering, would save on the sorting time. Nevertheless, the advantages of the sleeved system over hand loading are quite dramatic. Loading times are easily halved, and staff exposure to radiation was reduced similarly.

The TLD results are about four times higher than the time‐dose rate estimates, at least in the cases where they are comparable. Some inflation of the TLD readings is to be expected, since we used only cases that achieved in excess of the minimum detectable level of the dosimeter. The other cases, some of which had just as many seeds and similar loading times, gave ring badge readings below the 30‐mrem minimum. The lack of correlation between exposure and number of seeds was thought to be a result of monitoring and interpreting such low dose levels.

However, as an indicator of approximate dose, the TLD appeared to have performed quite well. The difference between the calculated and measured doses is reasonable given the caveats of the study environment. The TLD doses achieved tend to validate the results of the time‐dose rate model and gave a reasonable conversion from hand to whole body exposures.

## CONCLUSIONS

The time and exposure burden associated with the preparation of iodine loose seed implants has been characterized. Average conventional loading times are on the order of 50 min with a hand dose equivalent in excess of 10 mrem per case. Whole body exposures average about 1 mrem per case. Use of the sleeved loading system results in significant time savings, with preparation times less than half that of conventional methods. Exposure reductions of similar magnitude are also noted for the sleeved system.

## ACKNOWLEDGMENT

This work was supported in part by a grant from Imagyn Medical Technologies, Inc., Irvine, CA.
